# A Rare Case of Life-Threatening Hemorrhage

**DOI:** 10.7759/cureus.62878

**Published:** 2024-06-21

**Authors:** Mariam Soni, Juliet Penn

**Affiliations:** 1 Internal Medicine, Santa Barbara Cottage Hospital, Santa Barbara, USA; 2 Hematology and Oncology, University of California Los Angeles, Los Angeles, USA; 3 Hematology and Oncology, Santa Barbara Cottage Hospital, Santa Barbara, USA

**Keywords:** primary biliary cirrhosis, arteriovenous malformations, hemorrhage, factor viii, cirrhosis, acquired hemophilia

## Abstract

Acquired hemophilia A is a rare but severe autoimmune bleeding disorder that results from autoantibodies against clotting factor VIII (FVIII). Distinguishing acquired hemophilia from other more common causes of bleeding, such as chronic liver disease, disseminated intravascular coagulation, or sepsis-induced coagulopathies, is crucial in guiding the management of life-threatening hemorrhage. This study describes a patient with primary biliary cholangitis who was found to have acquired hemophilia A, a unique cause of life-threatening bleeding that was especially challenging to diagnose and manage with her underlying liver disease. Identifying acquired hemophilia A allowed an avenue of treatment options that would not have otherwise been available.

## Introduction

Acquired hemophilia A is a rare but severe autoimmune bleeding disorder that results from autoantibodies against clotting factor VIII (FVIII) [1]. While many cases are idiopathic, some are associated with malignancies, pregnancy, medications, or other autoimmune conditions as suspected in our patient’s case [1]. Autoantibodies to FVIII are the most common with an incidence of approximately 1 to 1.5 per million population per year, mostly arising in the sixth or seventh decade of life [2].

Individuals with chronic liver disease are also at risk for bleeding diathesis, and this comorbidity may complicate the diagnosis as in this case. The progression of cirrhosis leads to decreased synthetic function of the liver, causing impaired production of procoagulant factors excluding FVIII, anticoagulants, and thrombopoietin contributing to thrombocytopenia [3]. Furthermore, critically ill patients or those experiencing severe sepsis or septic shock may also develop bleeding from disseminated intravascular coagulation. While it may be easy to attribute life-threatening hemorrhage to underlying liver failure in a patient with cirrhosis, the importance of differentiating the etiology between liver disease and rare causes such as hemophilia is crucial in guiding management.

## Case presentation

The patient is an 80-year-old female with a history of cirrhosis (Child-Pugh Class C) secondary to primary biliary cholangitis (PBC) requiring biweekly paracentesis and acute on chronic blood loss anemia from duodenal arteriovenous malformations (AVM) requiring biweekly red blood cell transfusions who presented to the emergency department with fatigue and melena. She had been recently hospitalized a week before for a gastrointestinal bleed. Workup at that time included endoscopy which showed multiple duodenal AVMs with stigmata of recent bleeding, thus not amenable to cauterization. No varices were seen at this time.

She was afebrile with a heart rate of around 80 bpm and blood pressure of 100/40 mmHg. Physical examination was notable for pale skin, conjunctival pallor, a tender, distended abdomen with a fluid wave, scattered ecchymoses over forearms and left upper thigh, and rectal examination with black tarry stool. Labs were notable for a hemoglobin of 6.8 g/dL, with a baseline of around 8-9 g/dL, and elevated creatinine at 2.90 mg/dL compared to a baseline of 1.90 mg/dL. Activated partial thromboplastin time (aPTT) was elevated at 78.4 seconds and had increased from 35 seconds three months prior. International normalized ratio (INR) and prothrombin time (PT) were within normal limits (Table 1).

**Table 1:** Laboratory investigations. WBC: white blood cells; MCV: mean corpuscular volume; CO_2_: bicarbonate; BUN: blood urea nitrogen; AST: aspartate transaminase; ALT: alanine transaminase; ALP: alkaline phosphatase; PT: prothrombin time; INR: international normalized ratio; aPTT: activated partial thromboplastin time

Test	Observed value	Reference range
Complete blood count		
WBC	12.2 U/L	4.0-10.0 U/L
Hemoglobin	6.8 g/dL	13.0-17.0 g/dL
MCV	87.7 fL	82.4-100.9 fL
Platelets	147 U/L	150-450 U/L
Comprehensive metabolic panel		
Sodium	127 mmol/L	134-145 mmol/L
Potassium	6.2 mmol/L	3.5-5.1 mmol/L
Chloride	98 mmol/L	98-110 mmol/L
CO_2_	18 mmol/L	21-32 mmol/L
BUN	120 mg/dL	7-24 mg/dL
Creatinine	2.90 mg/dL	0.70-1.30 mg/dL
Albumin	2.0 g/dL	3.4-5.0 g/dL
Bilirubin	2.3 mg/dL	0.2-1.2 mg/dL
AST	42 U/L	<50 U/L
ALT	35 U/L	<61 U/L
ALP	133 U/L	42-122 U/L
Coagulation studies		
PT	11.1 seconds	10.1-13.7 seconds
INR	1.0	0.9-1.2
aPTT	78.4 seconds	26.7-40.1 seconds
Fibrinogen	127 mg/dL	200-400 mg/dL

She received transfusions of packed red blood cells with improvement in her hemoglobin and underwent a diagnostic paracentesis with 35 mL of clear yellow fluid removed. Due to discomfort from a distended abdomen, the following day she underwent large volume paracentesis with 5 L of bloody fluid removed, complicated by an abdominal wall hematoma and persistent bleeding with acute anemia requiring several more units of packed red blood cells, prothrombin complex concentrates, tranexamic acid, and desmopressin acetate (DDAVP). She was also treated with octreotide, norepinephrine, and albumin for suspected hepatorenal syndrome with octreotide additionally treating known AVMs.

With the isolated elevated aPTT, there was a concern for underlying hemophilia or a factor deficiency, so further workup was done. The aPTT mixing study did not correct, indicating the presence of an inhibitor, and FVIII activity was reduced at 1%. A Bethesda titer was obtained to quantify the strength of the inhibitor and was elevated at 18.5 BU/mL. Other studies done including factor VII (FVII) activity, protein C, protein S, iron studies, and vitamin B12 and folate were within normal limits (Table 2).

**Table 2:** Further investigation of coagulation studies. aPTT: activated partial thromboplastin time; vWF: von Willebrand factor; TIBC: total iron binding capacity

Test	Observed value	Reference range
aPTT mixing study		
aPTT	78.4 seconds	26.7-40.1 seconds
50/50 aPTT immediate (0 min) incubation	42.6 seconds	
50/50 aPTT 1 hour incubation	61.7 seconds	
Other coagulation studies		
Factor VIII activity	1%	56-140%
Bethesda titer	18.5 BU/mL	0.0-0.8 BU/mL
Factor VII activity	73%	51-186%
vWF antigen	512%	50-200%
vWF activity	522%	50-200%
Protein C	76%	73-180%
Protein S	70%	63-140%
Iron	64 ug/dL	50-170 ug/dL
TIBC	356 ug/dL	250-450 ug/dL
Iron saturation	25%	20-55%
Ferritin	74 ng/mL	11-306 ng/mL
Vitamin B12	690 pg/mL	180-914 pg/mL
Folate	17 ng/mL	2-20 ng/mL

Her hospital course was complicated by ongoing active melena. Repeat endoscopy was deferred given recently seen AVMs; a nuclear medicine GI bleed scan showed diffuse bleed throughout her proximal small bowel. A review with gastroenterology and interventional radiology did not show an area amenable to cauterization or embolization. She received antihemophilic factor recombinant FVIII, with some effect that diminished over the next few days, in addition to daily goal-directed transfusions of several units of packed red blood cells, cryoprecipitate, platelets, and prothrombin complex. She also received rituximab and high-dose prednisone as part of immunosuppressive therapy for acquired hemophilia. Amidst treatment of hepatorenal syndrome and ongoing bleeding and transfusion dependence, the patient and her family had multiple goals of care conversations and ultimately decided to pursue comfort-focused care. She passed away on the ninth day of hospitalization.

## Discussion

Acquired hemophilia A is important to consider as part of a differential diagnosis for severe bleeding especially in a patient with an isolated elevated aPTT. Comorbid conditions such as underlying end-stage liver disease and sepsis in a critically ill patient may confound the clinical picture when rare conditions such as acquired hemophilia may be the underlying culprit of life-threatening hemorrhage.

An elevated INR and PT with normal or near normal aPTT is often seen in cirrhosis, as opposed to an isolated elevated aPTT as in the patient in this case [4]. Isolated elevation in aPTT should lead to a mixing study to assess for a factor deficiency and presence of an inhibitor. Individual factors are tested, and if one is found to be low, a Bethesda titer is done to quantify the strength of the apparent inhibitor. A diagnosis of acquired hemophilia may lead to several avenues of treatment opportunities.

The goals of treatment in acquired hemophilia include achieving and maintaining hemostasis and eradicating the inhibitor. Agents that can be used to achieve hemostasis include recombinant porcine FVIII, bypassing agents such as recombinant FVIIa, or activated prothrombin complex concentrates. These agents may increase the risk of thrombosis, especially in a patient with chronic liver disease. Once hemostasis is achieved, eradication of the inhibitor is attempted with immunosuppression to re-establish FVIII immune tolerance [5].

Bleeding in the setting of cirrhosis, however, does not respond to treatments such as recombinant FVIIa, prothrombin complex concentrates, or tranexamic acid as it is often due to anatomic etiologies resulting from portal hypertension rather than dysregulation of hemostasis [6,7]. Patients often receive vitamin K in the setting of abnormal coagulation tests although the benefit is unclear and often does not lead to a significant reduction in PT or INR [8,9]. Prophylactic platelet or fresh frozen plasma transfusions are also often administered prior to procedural intervention. In variceal hemorrhage, patients often require endoscopic interventions or transjugular intrahepatic portosystemic shunt (TIPS) to decompress the portal system as salvage therapy.

While our patient initially set to receive further therapies including bypassing agents, this did not align with her goals of care and she ultimately passed away on comfort care. Recognizing another cause of bleeding in this patient-guided management in a direction that would not have otherwise been considered if her bleeding had been attributed solely to underlying cirrhosis. Although rare, it is crucial to consider acquired hemophilia in patients with an elevated aPTT and normal INR and PT, despite existing comorbid conditions that can complicate the clinical picture.

## Conclusions

Acquired hemophilia A is an important diagnosis to consider in a patient with isolated elevation of aPTT. Underlying comorbidities including cirrhosis can complicate the clinical picture but would often be seen with elevated INR and PT. A diagnosis of acquired hemophilia A, although rare, does have treatment options including bypassing agents and immunosuppressive therapy that can be considered to manage a life-threatening hemorrhage, maintain hemostasis, and eradicate the factor inhibitor.
